# Treatment Patterns and Outcomes of Stage III Melanoma Patients with Positive Sentinel Lymph Node Biopsy: A Real-Life Experience

**DOI:** 10.3390/jcm13175238

**Published:** 2024-09-04

**Authors:** Gabriele Roccuzzo, Nicole Macagno, Pietro Grignani, Chiara Astrua, Matteo Giovanni Brizio, Giovanni Cavaliere, Franco Picciotto, Virginia Caliendo, Enrico Fruttero, Simone Ribero, Paolo Fava, Pietro Quaglino

**Affiliations:** 1 Department of Medical Sciences, Section of Dermatology, University of Turin, 10126 Turin, Italy; nicole.macagno@edu.unito.it (N.M.); pietro.grignani@edu.unito.it (P.G.); chiaraastrua@gmail.com (C.A.); matteobrizio@virgilio.it (M.G.B.); g.cavali_dr@yahoo.it (G.C.); simone.ribero@unito.it (S.R.); fava_paolo@yahoo.it (P.F.); pietro.quaglino@unito.it (P.Q.); 2 Department of Surgery, Dermatologic Surgery Section, Azienda Ospedaliera Universitaria (AOU) Città della Salute e della Scienza, 10126 Turin, Italy; franco.picciotto@gmail.com (F.P.); virginiacaliendo@gmail.com (V.C.); enrico.fruttero@unito.it (E.F.)

**Keywords:** melanoma, lymphadenectomy, lymph node dissection, stage III melanoma, adjuvant therapy, immunotherapy, targeted therapy

## Abstract

**Background:** Advancements in managing stage III melanoma have involved the implementation of adjuvant therapies alongside a simultaneous decrease in the utilization of completion lymph node dissection (CLND) following positive sentinel node biopsy (SLNB). **Methods:** This retrospective study from the University of Turin’s Dermatology Clinic analyzed relapse-free survival (RFS) and overall survival (OS) among stage III melanoma patients (n = 157) who underwent CLND after positive SLNB versus those who did not receive such procedure. **Results:** Patients without CLND had a median RFS of 49 months (95% CI 42-NA), while CLND recipients showed 51 months (95% CI 31-NA) (*p* = 0.139). The 48-month OS for non-CLND patients was 79.8% (95% CI 58.2–91.0) versus 79.2% (95% CI 67.5–87.0) for CLND recipients (*p* = 0.463). Adjusted Hazard Ratios through inverse probability treatment weighting revealed the impact of CLND to be insignificant on RFS (aHR 0.90, 95% CI 0.37–2.22) and marginal on OS (aHR 0.41, 95% CI 0.13–1.21). Conversely, adjuvant therapy significantly reduced the risk of relapse (aHR 0.46, 95% CI 0.25–0.84), irrespective of CLND. **Conclusions**: This study corroborates the growing evidence that CLND after positive SLNB does not enhance RFS or OS, while emphasizing the crucial role of adjuvant therapy, be it immunotherapy or targeted therapy, in reducing the risk of relapse in melanoma patients with positive SLNB.

## 1. Introduction

The incidence of melanoma has been increasing worldwide, showing an annual increase of about 5% [1]. Current standard treatment typically involves wide local excision of the primary tumor alongside sentinel node biopsy (SNLB) for tumors with a Breslow thickness of ≥1.0 mm or ≥0.8 mm with additional risk factors, like ulceration [2]. This staging procedure is considered suitable for patients in whom neither palpation nor lymph node sonography hints at lymph node metastases (macro-metastases), with SLNB involvement independently impacting melanoma-specific survival [3]. Over the past decade, three pivotal changes have significantly altered the landscape of melanoma management: the introduction of the 8th Edition of the American Joint Committee on Cancer (AJCC) staging system in 2017, the evolving insights from clinical trials regarding completion lymph node dissection (CLND) in SLNB-positive patients, and the integration of adjuvant therapy into real-life practice [2,4,5]. The AJCC staging system incorporates tumor thickness (T), lymph node involvement (N), and the presence of distant metastases (M) into TNM classification. The 8th edition introduced significant changes, particularly in stage III, with patients with tumors ≥0.8 mm Breslow index now indicated for SNLB, alongside the introduction of stage IIID [6]. Moreover, clinical trials have provided transformative insights into the management of regional lymph nodes in melanoma patients. The introduction of SLNB marked a significant milestone, with pioneering results presented in 1990 [7]. Subsequent trials such as the Multicenter Selective Lymphadenectomy Trial I (MSLT-I), German Dermatologic Cooperative Oncology Group Selective Lymphadenectomy Trial (DeCOG-SLT), and Multicenter Selective Lymphadenectomy Trial II (MSLT-II) have guided clinical decision-making [8]. Specifically, MSLT-I established the pivotal role of SLN biopsy in determining prognosis in melanoma, leading to improved regional disease control. However, it did not reveal a melanoma-specific survival benefit for the overall patient population [9]. In the multicentric DeCOG-SLT randomized phase 3 trial, the survival outcomes of SLN-positive melanoma patients with and without CLND were compared [10]. Surprisingly, there was no discernible difference in survival between the two groups, suggesting that CLND might not be warranted in patients with sentinel node micrometastases of 1 mm or less in diameter. Similarly, MSLT-II findings indicated no significant disparity in melanoma-specific survival despite the enhanced regional lymph-node control achieved through dissection [11]. Meanwhile, the pivotal phase III randomized clinical trials including Keynote-054, CheckMate-238, and COMBI-AD have validated the efficacy of adjuvant therapy regimens in resected stage III melanomas, comprising immunotherapy (IT) or targeted therapy (TT) in BRAF-mutant melanomas [12,13,14,15]. Significant improvement in terms of RFS (relapse-free survival) was reported with nivolumab (5-year RFS 50% vs. 39% for ipilimumab), pembrolizumab (5-year RFS 55.4% vs. 38.3% for placebo), and the combination of dabrafenib and trametinib (5-year RFS: 52% vs. 36% for placebo) [13,16,17]. However, disparities between clinical trial outcomes and real-life clinical practice are evident. While pivotal studies were conducted under the previous AJCC 7th classification and involved CLND post-positive SNLB, real-world patients are now staged according to the AJCC 8th classification, with only a minority undergoing CLND. This discrepancy underscores the need for further investigation into the role of CLND and its interplay with adjuvant therapy in real-world patient cohorts. In the present paper, we investigate the role of CLND after a positive SNLB and its association with adjuvant therapy in a cohort of real-life patients. 

## 2. Materials and Methods

This study was approved by Comitato Etico Interaziendale AOU Città della Salute e della Scienza di Torino (TESEO—0061280) and was conducted in accordance with the principles of the declaration of Helsinki. A retrospective series of melanoma patients with positive SLNB evaluated at the Dermatology Clinic of the Turin University Hospital, Italy, between January 2017 and December 2022 were collected. All patient information was sourced from the hospital’s database and subsequently archived within an internal computerized database. Patients’ inclusion criteria were age ≥18 years, histologically confirmed diagnosis of melanoma, and a confirmed stage III (A, B, C, and D) according to the AJCC 2017 (TNM 8th edition) after histological confirmation of a metastasis in SLN [6]. The absence of distant metastasis was assessed prior to SNLB in all patients by total-body CT scans or PET-CT plus brain MRI. CLND subsequent to a positive SLNB was determined on an individual basis by the tumor board [10,11]. The selection of the adjuvant regimen occurred in a multidisciplinary setting, adhering to local prescribing policies and factoring in BRAF status and patients’ comorbidities. Specifically, adjuvant therapy became the standard of care in Italy towards the close of 2019, as prior to that, only a few patients had undergone it within the framework of clinical trials and expanded access programs. The adjuvant regimen included targeted therapy (TT) with dabrafenib trametinib (300 mg + 2 mg/day) for BRAF-mutant patients or immunotherapy (IT) with either nivolumab (240 mg every two weeks or 480 mg every 4 weeks) or pembrolizumab (200 mg every 3 weeks or 600 mg every 6 weeks) regardless of the BRAF status. The therapy lasted until the completion of the 12-month cycle unless there was disease progression or unacceptable toxicity. Study endpoints were the following: relapse-free survival (RFS), as the time from the start of therapy to the date of the first recurrence or death from any cause; overall survival (OS), as the time from the start of therapy until death. For patients alive without disease recurrence or metastasis development, data were censored on the date of last patient contact. Descriptive statistics were used for patient and tumor characteristics. Mann–Whitney, Chi-squared with Yates corrections, and Fisher’s exact tests were used to analyze continuous and paired nominal data, respectively. To address confounding due to the lack of randomization, regression analysis was employed to manage potential imbalances between treatment groups. Diagnostics through variance inflation factor (VIF) were used to rule out multicollinearity among independent variables. Model fitness was evaluated according to McFadden’s formula. The proportional-hazards assumption on the basis of Schoenfeld residuals was tested, and multivariable Cox regression models were used to simultaneously adjust for baseline characteristics [18]. Baseline covariates that could cause concern if imbalances existed were selected a priori (stage IIIA, IIIB, IIIC, and IIID; age; sex; Breslow thickness; ulceration; and adjuvant therapy). Analyses were restricted to independent variables with data available for over 75% of the cohort, following common practice. Survival curves were generated based on the Kaplan–Meier method and analyzed through the Log-rank test. Using an inverse-probability-of-treatment weights (IPTW) approach, we computed the propensity scores for CLND to estimate the marginal HR for CLND and marginal survival curves [18]. Patients were censored at the time of last follow-up. A *p*-value of ≤0.05 was considered statistically significant. All statistical analyses were performed using Stata/SE.v.18 Software (StataCorp, College Station, TX, USA).

## 3. Results

### 3.1. Population Description

The analysis encompassed a cohort of 157 patients, with Table 1 summarizing the baseline characteristics. The overall median follow-up for the cohort was 36 months (range 3–90). Among the patients, 88 (56.1%) underwent CLND, while 69 (44.9%) did not. The baseline demographic features between these groups showed no significant differences, with comparable mean age and sex distribution. Regarding melanoma features, both groups exhibited similar patterns. The trunk was the predominant site in both, constituting 43.5–55.7% of cases, followed by the lower limbs, at 22.7–33.3%, and upper limbs, at 10.1–14.8%. The most common histological subtypes were superficial spreading melanoma (SSM) (42–43.2%) and nodular melanoma (27.5–30.7%). No noteworthy differences were observed in terms of Breslow thickness, vertical growth patterns, ulceration, number of mitoses, and presence of either perineural or lymphovascular invasion. In terms of SLNB, both groups showed comparable mean numbers of SNLs evaluated, maximum diameter of lymph node metastasis, and metastatic site feature within the lymph node. BRAF mutation prevalence was consistent in both groups, ranging from 43.2% to 50.7%. The subsequent administration of adjuvant therapy was not influenced by baseline tumor characteristics but rather by chronological differences. Notably, 97.1% of patients without CLND and 75.0% of those with CLND received adjuvant therapy (*p* = 0.001). Hence, 25.0% of the members of the latter group were treated before the approval of such regimens or were not included in any expanded-access program. As for those receiving adjuvant therapy, there was a similar distribution of targeted therapies (57.6–52.4%) and immunotherapy (42.4–47.8%) between the two groups. 

### 3.2. Survival Analysis: Relapse-Free Survival

**Figure 1:**
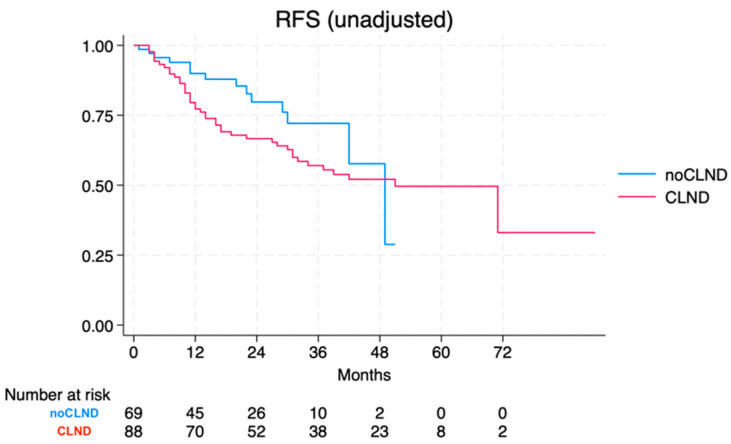
Relapse-free survival of the no-CLND vs. the CLND cohort.

At the time of data cut-off, a total of 56 events were observed. The median RFS for patients without CLND was 49 months (95% CI 42-NA), while for those who underwent CLND, it was 51 months (95% CI 31-NA). The log-rank test yielded a *p*-value of 0.139. For CLND, the crude HR was 1.58 (95% CI 0.85–2.93) (Figure 1).

**Figure 2:**
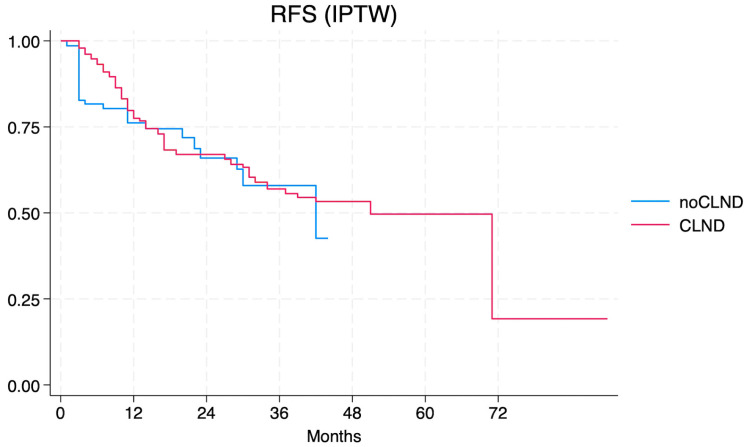
Relapse-free survival according to the IPTW model of the no-CLND vs. the CLND cohort.

After applying the IPTW model, the aHR for CLND dropped to 0.90 (95% CI 0.37–2.22), without achieving statistical significance (Figure 2). 

In the context of the Cox regression univariate analysis, various factors displayed associations with the relapse outcome. On the entire cohort, adjuvant therapy demonstrated a protective effect, with an HR of 0.47 (95% CI 0.25–0.87, *p* = 0.016). Stage IIIA exhibited a protective effect, with an HR of 0.24 (95% CI 0.09–0.67, *p* = 0.006), whilst a worse prognosis was recorded in stages IIIC and IIID, showing HRs of 4.72 (95% CI 1.67–13.28, *p* = 0.003) and 6.91 (95% CI 1.82–26.31, *p* = 0.005), respectively. Other significant negative factors included Breslow thickness (HR 1.09, 95% CI 1.02–1.16, *p* = 0.009), ulceration (HR 4.57, 95% CI 2.29–9.12, *p* < 0.001), number of mitoses (HR 1.08, 95% CI 1.02–1.15, *p* = 0.008), lymphovascular invasion (HR 1.91, 95% CI 1.02–3.59, *p* = 0.043), number of positive sentinel lymph nodes (HR 2.15, 95% CI 1.15–4.02, *p* = 0.016), maximum diameter of lymph node metastasis (HR 1.11, 95% CI 1.01–1.22, *p* = 0.046), extracapsular extension (HR 8.94, 95% CI 2.62–30.48, *p* < 0.001), SSM histology (HR 2.12, 95% CI 1.15–3.91, *p* = 0.016), and nodular histology (HR 5.5, 95% CI 1.68–18.1, *p* = 0.005). Conversely, lentigo maligna melanoma histology was associated with a reduced risk of relapse (HR 0.45, 95% CI 0.25–0.79, *p* = 0.006). The further subgroup analysis of the different groups of the study revealed some other specific associations. In the no-CLND group, stage IIID (HR 8.83, 95% CI 1.04–76.31, *p* = 0.048), Breslow thickness (HR 1.20, 95% CI 1.06–1.38, *p* = 0.006), ulceration (HR 5.05, 95% CI 1.12–22.86, *p* = 0.035), number of mitoses (HR 1.21, 95% CI 1.03–1.46, *p* = 0.020), tumor-infiltrating lymphocytes (TILs) (HR 0.06, 95% CI 0.01–0.31, *p* = 0.001), and tumor site on the trunk (HR 0.08, 95% CI 0.01–0.68, *p* = 0.020) were significantly associated with relapse. In the CLND group, ulceration (HR 4.9, 95% CI 2.14–11.18, *p* < 0.001) and nodular melanoma (HR 4.25, 95% CI 1.27–14.21, *p* = 0.019) were strongly associated with the risk of relapse, whilst stage IIIC (HR 1.91, 95% CI 1.01–3.63, *p* = 0.047), lentigo maligna melanoma histology (HR 0.51, 95% CI 0.26–0.99, *p* = 0.048), and BRAF mutation (HR 0.35, 95% CI 0.13–0.92, *p* = 0.034) showed a weaker association. Table 2 depicts the selected multivariate model, incorporating the aforementioned variables based on the frequency of relapse events. 

### 3.3. Survival Analysis: Overall Survival

**Figure 3:**
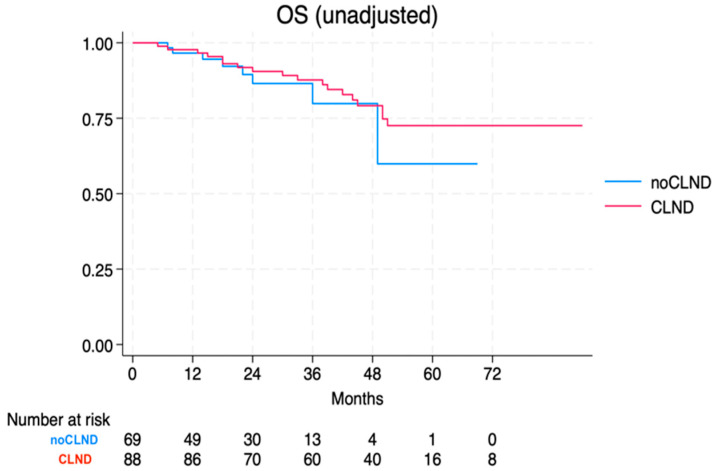
Overall survival of the no-CLND vs. the CLND cohort.

At the time of data cut-off, a total of 26 deaths were recorded. Patients without CLND exhibited an unreached median overall survival (OS), with a 48-month survival rate of 79.8% (95% CI 58.2–91.0). Likewise, for those undergoing CLND, the median OS was unreached, with a 48-month survival rate of 79.2% (95% CI 67.5–87.0). The log-rank test showed no significant difference, with a *p*-value of 0.463. For CLND, the crude HR was 0.72 (95% CI 0.29–1.74) (Figure 3).

**Figure 4:**
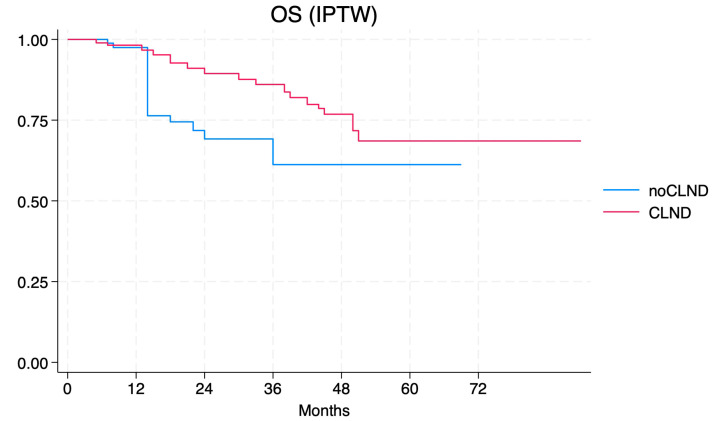
Overall survival according to the IPTW model of the no-CLND vs. the CLND cohort.

After applying the IPTW model, the aHR for CLND dropped to 0.41 (95% CI 0.13–1.21), without achieving statistical significance (Figure 4). 

In the Cox regression univariate analysis, various factors displayed significant associations with the survival outcome. Distant relapse markedly revealed a substantial HR of 18.84 (95% CI 4.45–79.81, *p* < 0.001), followed by loco-regional relapse, with an HR of 6.05 (95% CI 2.79–13.14, *p* < 0.001). Additionally, stage IIIC demonstrated an HR of 7.81 (95% CI 1.05–58.26, *p* = 0.045), while Breslow thickness and ulceration exhibited HRs of 1.09 (95% CI 1.01–1.19, *p* = 0.036) and 6.57 (95% CI 1.97–21.9, *p* = 0.02), respectively. Lymphovascular invasion and the number of positive sentinel lymph nodes also showed significant HRs of 2.58 (95% CI 1.02–6.55, *p* = 0.046) and 2.73 (95% CI 1.42–5.25, *p* = 0.003), respectively.

Further scrutinizing the different groups, we noted that patients who did not undergo CLND displayed Breslow thickness (HR 1.27, 95% CI 1.10–1.48, *p* = 0.001), lymphovascular invasion (HR 14.4, 95% CI 1.66–124.15, *p* = 0.015), the number of sentinel SLN (HR 2.31, 95% CI 1.14–4.66, *p* = 0.020), and locoregional progression (HR 15.33, 95% CI 3.58–65.52, *p* < 0.001) as significant variables associated with the risk of death. Notably, superficial spreading melanoma (SMM) demonstrated an HR of 5.91 (95% CI 1.05–33.45, *p* = 0.044).

In contrast, within the CLND group, significant factors included stage IIIC (HR 3.44, 95% CI 1.13–10.48, *p* = 0.030), ulceration (HR 4.50, 95% CI 1.30–15.58, *p* = 0.018), and visceral melanoma (HR 16.4, 95% CI 1.91–140.51, *p* = 0.011). Distant progression exhibited a noteworthy HR of 10.83 (85% CI 2.49–47.1, *p* = 0.001), while locoregional relapse had an HR of 4.69 (95% CI 1.83–12.01, *p* = 0.001). Table 3 depicts the selected multivariate model, incorporating the aforementioned variables based on the frequency of relapse events.

### 3.4. Patients Outcomes

**Figure 5:**
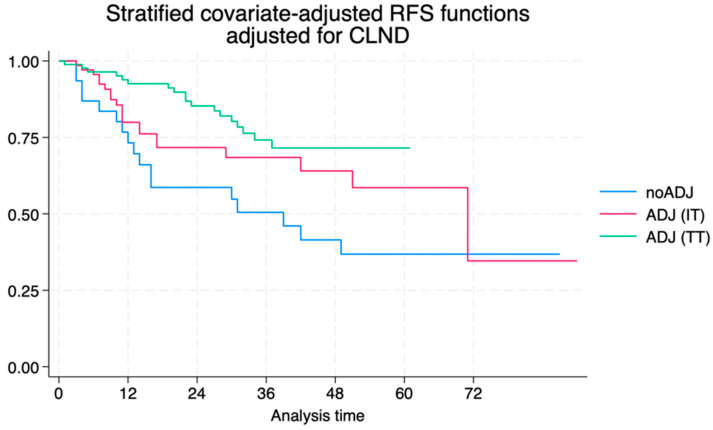
Adjusted relapse-free survival curves according to adjuvant therapy.

The examination of factors influencing the choice to undergo CLND in melanoma patients revealed no significant difference related to melanoma stage. Specifically, the decision to undergo CLND was essentially influenced by the timing, occurring either before or after 2019, and was not contingent on the initial melanoma stage following a positive SLNB. Moreover, patients undergoing adjuvant therapy were significantly less likely to have received CLND (*p* = 0.001), suggesting a shift in clinical practice where adjuvant therapy was prioritized over CLND. In an event-rate analysis, the choice of dissection itself was not found to be protective against the risk of local or distant relapse, with similar rates in both groups of patients. Specifically, sole locoregional relapse accounted for 35.7% (15/42) and 28.9% (4/14) of the relapses in the CLND and no-CLND groups, while distant relapse with/without local relapse was observed in the other 64.3% (27/42) and 71.4% (10/14), respectively. Contrastingly, the most significant benefit in terms of relapse risk reduction was associated with adjuvant therapy (Figure 5). In fact, patients who underwent adjuvant therapy were less likely to experience relapse compared to those not receiving it, regardless of the CLND procedure and BRAF status, with no significant differences between the IT and TT regimens (aHR for adjuvant therapy: 0.46, 95% CI 0.25–0.84). 

Overall, out of 60 patients receiving immunotherapy, 20 experienced relapse (33%), with 8 sole locoregional (40%) and 12 distant relapses (60%), whilst out of 73 patients receiving targeted therapy, 18 experienced relapses (24.6%), with 6 sole locoregional (33.3%) and 12 distant relapses (66.7%) (*p* = 0.270). 

**Figure 6:**
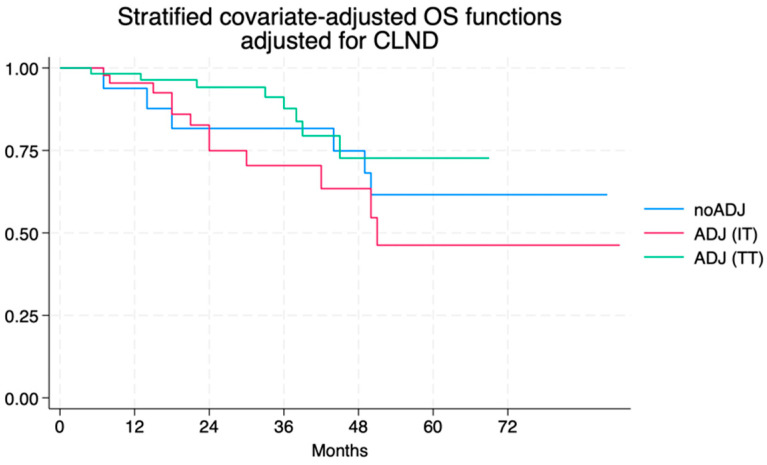
Adjusted overall survival curves according to adjuvant therapy.

This benefit in risk-of-relapse reduction did not translate into an appreciable difference in overall survival between patients receiving adjuvant therapy and those who did not (aHR for adjuvant therapy: 1.00, 95% CI 0.39–2.69, Figure 6)

## 4. Discussion

In the past decade, the management of stage III melanoma has undergone a significant transformation, characterized by noteworthy shifts. These changes encompass the widespread adoption of adjuvant therapies as standard practice and the revelation from clinical trials that conducting CLND in cases of positive SLNB does not influence survival [10,11,19,20]. Hence, an international cohort study conducted across 21 melanoma centers from 2017 to 2019 documented a reduction in CLND and a rise in the utilization of adjuvant systemic therapy among SLN-positive melanoma patients, with variations in practice influenced by factors such as tumor size, disease stage, and center location [20]. Despite this overarching trend, disparities in patient demographics and care settings significantly contribute to the variability in treatment trends [21,22,23,24]. Therefore, validating the findings from clinical trials in real-world scenarios remains essential, especially given the need for caution when comparing studies and settings. Consequently, our study addresses this necessity by investigating a real-life cohort of stage III melanoma patients with positive SLNB, excluding those with clinically or imaging detectable metastatic lymph nodes. Our study confirms that the choice of CLND was primarily influenced by timing, with a reduced inclination for dissection after the outcomes of the DeCOG and MSLT-2 trials, along with an increase in adjuvant therapy prescription based on drug availability [7,8,10]. Overall, several key points emerged. Firstly, there was no discernible difference in terms of RFS between the CLND and no-CLND groups, both in terms of loco-regional and distant metastasis. Secondly, overall survival outcomes did not significantly differ, aligning with the results of clinical trials [7,8,10]. The initial worse trend observed in the IPTW model around 12–24 months for the no-CLND group likely highlights the latent effects previously masked by the protective impact of adjuvant therapy in this group. This adjustment uncovers the potential impact of CLND once the confounding effect of adjuvant therapy is controlled for. Third, the administration of adjuvant therapy emerged as the main protective measure against relapse, halving the risk irrespective of the CLND procedure. However, such benefits did not translate into an appreciable improvement in the OS, likely due to the short follow-up [25,26]. Furthermore, several findings regarding prognostic factors have shown interesting results. The analysis revealed that ulceration status was significantly linked to the risk of relapse, while the lymph-node tumor deposit showed a weaker association in the univariate model [27]. As expected, lentigo maligna melanoma was protective compared to other histological subtypes. For OS, distant relapse had the strongest association with the outcome, outweighing locoregional relapse. Our findings align with evidence from other real-life studies. According to Palve et al., a tumor deposit diameter > 4 mm with multifocal SN distribution was a key predictor of prognosis, suggesting adequate patient stratification and risk classification based on these parameters [28]. Similarly, the multicentric experience from the Italian Melanoma Intergroup identified older age, male gender, increasing Breslow thickness, presence of ulceration, larger sentinel node tumor burden size, and metastatic non-sentinel nodes as independent negative predictors of survival, suggesting that prognosis decays with increasingly higher larger metastatic deposit within the sentinel node [29]. Regarding OS, locoregional relapse significantly heightened the risk of death in our study, yet these data need to be analyzed in context. While locoregional relapse occurred equally among patients who underwent CLND and those who did not, it was more prevalent in patients who did not receive adjuvant therapy. These observations suggest that the lower risk of locoregional relapse may be related to the adjuvant therapy itself rather than the CLND procedure. Such findings are in line with similar results from other recent studies. In 2023, a multicentric Polish study including a cohort of 147 melanoma patients treated at eight centers with adjuvant therapy reported that the type of lymph node surgery before adjuvant therapy did not influence the outcomes, and CLND after positive SLNB did not affect the results in terms of RFS or OS [30]. Similarly, in the study by Quildrian et al., no differences in terms of 2-year MSS and DMFS were found between active surveillance and CLND groups in SNLB+ patients [20]. Another multicentric study including seven cancer centers with a median follow-up of 25 months showed that the percentage of patients undergoing CLND decreased between 2017 and 2021, while the use of concomitant adjuvant treatment increased [31]. At 3 years, adjuvant therapy prolonged RFS (HR:0.69, *p* = 0.036), but CLND did not (HR:1.22, *p* = 0.272), with no statistically significant differences in OS for either adjuvant systemic treatment or CLND. Similar trends were seen in a Japanese experience, despite different genetic and histological features, with adjuvant therapy tending to prolong a patient’s RFS, while omitting immediate CLND had no significant negative influence on it [32]. In another investigation led by Eroglu et al., it is suggested that providing adjuvant immunotherapy might offer comparable efficacy in SLNB+ patients who choose not to undergo immediate CLND, as salvage surgery, specifically therapeutic lymphadenectomy upon relapse, could serve as a feasible option in those cases of later locoregional relapse [33]. At last, in BRAF-mutant patients receiving adjuvant therapy, both immunotherapy and targeted therapy regimens exhibited equal effectiveness in reducing the risk of relapse and death, bringing new evidence to the current debate [34]. Overall, our study confirms the similar trends in terms of RFS and OS in patients with positive SNLB followed by CLND or follow-up and further highlights the value of adjuvant therapy in reducing the risk of relapse. 

Overall, some study limitations need acknowledgment. Firstly, one of the primary limitations of this study is the small sample size and the limited follow-up period, resulting in sparse data, particularly after 36 months. This issue is most pronounced in the non-CLND group, with limited data at later time points limiting the study’s ability to make definitive statements about long-term relapse-free and overall survival. Second, reliance on data from a single center may limit the generalizability of findings to broader populations. Third, the retrospective design inherently introduces constraints in data collection. Incomplete data necessitated conducting regression analyses only on variables available for the majority of patients, which precluded assessing certain recognized prognostic factors like LDH values or S-100 [35]. Additionally, the study included a minority of patients with melanoma of the head and neck, where the role of CLND is more debated [36,37,38]. Despite these limitations, our findings contribute to the ongoing discussions about treatment strategies and emphasize the need for further research to refine therapeutic approaches and improve patient outcomes in this complex clinical context [39]. 

## 5. Conclusions

Our findings indicate a diminishing relevance of CLND in melanoma patients testing positive for SLNB, showing no significant influence on relapse or mortality reduction. Adjuvant therapy, encompassing targeted or immunotherapies, substantially diminishes relapse risk in such individuals and should be therefore offered to all SLNB-positive patients, irrespective of CLND status. Further studies are welcomed to assess the implementation of effective prognostic biomarkers in guiding patient selection for CLND and adjuvant therapy.

## Figures and Tables

**Table 1:** Study population.

	CLND (n = 88)	No-CLND (n = 69)	*p*-Value
Age, mean (95% CI)	57.3 (53.0–60.3)	56.7 (54.5–59.9)	0.833
Sex, male (%)	50 (56.8%)	35 (50.1%)	0.506
Stage, n° (%)	IIIA: 12 (13.6%)	IIIA: 20 (28.9%)	0.112
	IIIB: 24 (27.3%)	IIIB: 15 (21.8%)	
	IIIC: 47 (53.4%)	IIIC: 32 (46.4%)	
	IIID: 5 (5.7%)	IIID: 2 (2.9%)	
Melanoma site, n° (%)	Head-Neck: 4 (4.5%)	Head-Neck: 6 (8.7%)	0.201
	Upper limbs: 13 (14.8%)	Upper limbs: 7 (10.1%)	
	Trunk: 49 (55.7%)	Trunk: 30 (43.5%)	
	Lower limbs: 20 (22.7%)	Lower limbs: 23 (33.3%)	
	Visceral: 1 (1.1%)	Visceral: 3 (4.3%)	
	NA: 1 (1.1%)	NA: 0 (0%)	
Histological type, n° (%)	SSM: 38 (43.2%)	SSM: 29 (42.0%)	0.447
	Nodular: 27 (30.7%)	Nodular: 19 (27.5%)	
	LMM: 0 (0%)	LMM: 2 (2.9%)	
	ALM: 5 (5.7%)	ALM: 6 (6.7%)	
	Nevoid: 0 (0%)	Nevoid: 1 (1.4%)	
	Mucosal: 1 (1.1%)	Mucosal: 2 (2.9%)	
	Desmoplastic: 1 (1.1%)	Desmoplastic: 0 (0%)	
	NA: 16 (18.1%)	NA: 19 (26.0%)	
Breslow, mean (95% CI)	4.4 mm (3.6–5.1)	4.1 mm (3.2–4.9)	0.677
Vertical growth, present (%)	33 (37.5%)	29 (42.0%)	0.564
Ulceration, present (%)	47 (53.4%)	34 (49.3%)	0.672
N° mitosis, mean (95% CI)	6 (5–7)	6 (5–7)	0.763
Perineural invasion, present (%)	3 (3.4%)	3 (4.3%)	0.848
Lymphovascular invasion, present (%)	20 (22.7%)	15 (21.7%)	0.701
Sentinel lymph nodes evaluated, n°	2 (1–3)	2 (1–3)	0.539
Maximum diameter of metastasis in the lymph node, mean (95% CI)	3.5 mm (2.6–4.3)	2.1 mm (1.3–2.7)	0.995
SLN metastasis site, n° (%)	Subcapsular: 11 (12.5%)	Subcapsular: 29 (42.0%)	0.163
	Parenchymal: 5 (5.7%)	Parenchymal: 8 (11.6%)	
	Mixed: 21 (23.9%)	Mixed: 23 (33.3%)	
	NA: 51 (57.9%)	NA: 9 (13.0%)	
BRAF mutant, n° (%)	38 (43.2%)	35 (50.7%)	0.381
Adjuvant therapy, n° (%)	66 (75.0%)	67 (97.1%)	0.001
	Targeted therapy: 38 (57.6%)	Targeted therapy: 35 (52.4%)	
	Immunotherapy: 28 (42.4%)	Immunotherapy: 32 (47.8%)	
Years of treatment			
2017–2019	57 (64.8%)	14 (20.3%)	<0.001
2020–2022	31 (35.2%)	55 (79.7%)	

**Table 2:** Multivariate Cox regression on RFS.

Variable	HR	95% CI	*p*-Value
CLND	1.23	0.61–2.45	0.551
Adjuvant therapy	0.53	0.27–1.02	0.059
Age	1.00	0.98–1.03	0.655
Male sex	0.69	0.39–1.20	0.188
Stage (IIIA reference)			
IIIB	2.19	0.69–7.01	0.183
IIIC	3.66	1.25–10.71	0.018
IIID	5.89	1.52–22.75	0.010

**Table 3:** Multivariate Cox regression on OS.

Variable	HR	95% CI	*p*-Value
CLND	0.55	0.23–1.28	0.167
Stage IIIC	2.32	0.92–5.81	0.071
Distant progression	17.58	4.10–75.25	<0.001

## Data Availability

Upon reasonable request to the corresponding author.
